# Small-scale eye care research: why and how to do it

**Published:** 2023-01-30

**Authors:** Esmael Habtamu, Priya Morjaria, Suzanne Gilbert

**Affiliations:** Assistant Professor in Eye Health: International Centre for Eye Health, London School of Hygiene & Tropical Medicine, London, UK and Chief Executive Director: Eyu-Ethiopia Eye Health Research, Training and Service Centre, Bahirdar, Ethiopia.; Assistant Professor and Public Health Optometrist: London School of Hygiene & Tropical Medicine and Head of Global Programme Design: Peek Vision, UK.; Senior Director, Research & Strategic Opportunities Seva Foundation, Berkeley, CA, USA

## Abstract

In resource-limited settings, small-scale research can focus on community-specific development needs and provide answers to context-specific challenges through pragmatic enquiry and data synthesis.

**Figure:**
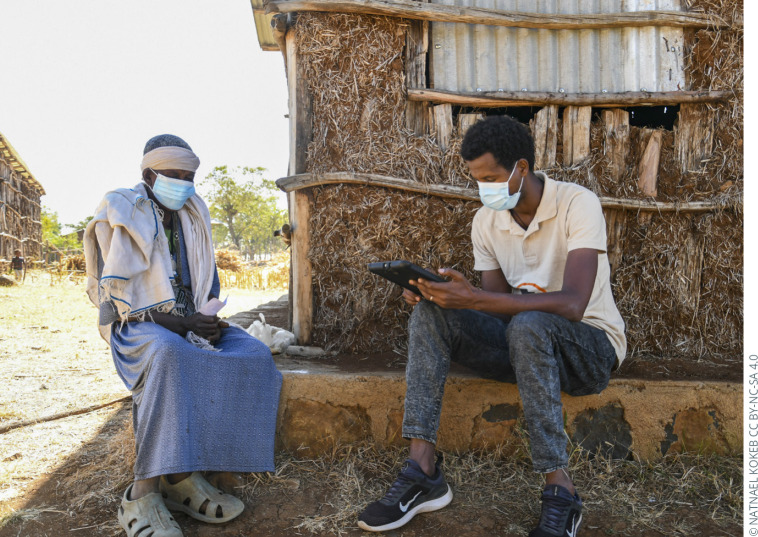
Collecting data through a community-based interview ethiopia

Research is a systematic investigation of new or existing concepts, methodologies, and understanding. This systematic investigation can be done on a large or small scale. Table 1 summarises key differences between large- and small-scale research. Large-scale research is often needed for questions that require greater statistical reliability and generalisability. However, in resource-limited settings, the barriers to conducting large-scale research are often the financial and human resources required. Small-scale research, on the other hand, focuses on providing answers to context-specific challenges with limited resources, and does so through pragmatic enquiry and data synthesis. For instance, through the Integrated Management of Presbyopia in Rural Ethiopia (IMPiRE) study, we have piloted the feasibility and acceptability of integrated presbyopia management by primary health care workers as part of the routine primary health care (PHC) system, in four PHC facilities, with a very limited budget.

**Table 1:** Key differences between large- and small-scale research

**Criteria**	**Large-scale research**	**Small-scale research**
Research question	Theory evaluation or testing intervention; often relevant to the wider discipline	Typically, relevant to local and context-specific challenges
Design	Focus on greater statistical reliability and generalisability	Flexible design with the focus on benefits to the local organisation or community
Ethics	Adherence to the appropriate ethical codes and guidelines	Adherence to the appropriate ethical codes and guidelines, plus consideration of any locally sensitive issues
Data collection	Requires separate, often complex, data collection tools and processes	Can be easily embedded within existing facility or community-based data collection
Resources	Costly in terms of time and money	No or minimal cost
Scale	Involves a large number of researchers, participants, and geographic areas or multiple sites	Conducted within the organisation or confined to a limited area with a small team of researchers and participants
Data measurement	Focuses on statistical or monetary standards	Focuses on producing manageable recommendations
Data interpretation	Both correlation and causality are possible, depending on the design	Needs greater caution; no causality can be inferred
Review	Peer review in publications	Collaborative review with stakeholders

The focus of small-scale research is usually not generalisability or publication; rather, it is developmental, with the aim of making recommendations to address a particular challenge or to improve services.^1^ Here we discuss some of the important aspects of conducting small-scale research.

## Developing a scientific mindset

A common limiting factor in conducting small-scale research is the assumption that research has to be a complex and expensive undertaking. However, the quality of research doesn't depend upon its size or the resources it requires. Research can be simple, robust, and pragmatic. It can be conducted at minimal or no cost but be impactful. Developing a scientific mindset and culture is a vital starting point.

A scientific mindset is characterised by curiosity, open-mindedness, and scepticism.^2^ Curiosity is constantly asking questions about why something works or doesn't work, how can it be improved, how a challenge can be addressed, and what tools are required to do this. For instance, in the IMPiRE pilot study, we were curious about whether we could improve the accessibility of presbyopia services for rural resource-limited communities.

Open-mindedness means to consider, at the beginning, that all possibilities are valid – until they are disproved. There should not be a predetermined idea of what works and what doesn't work until tested. In the IMPiRE study, we were ready for any possible result: for example, that the community and health service managers would either accept or not accept the delivery of presbyopia services by PHC workers.

However, such open-mindedness should also include systematic doubt or scepticism. As the research progresses, it is important to question procedures and results. This is related usually to questioning the quality of the research or the data produced. In the IMPiRE study, we needed to be clear about the procedure we used to conduct the study, and its limitations, so that the results could be interpreted within defined and sensible limits.

Curiosity, open-mindedness, and scepticism are linked, respectively, with developing the research question, formulating methodologies, and interpreting the results.

## Research question

The first step in conducting any research is identifying the research question or challenge that needs to be investigated. In small-scale research, the questions that are addressed are those that are typically relevant to the local context. Such questions often arise from curiosity about our clinical practices, engagement with stakeholders and the community, a review of medical records or reports, and questioning of the productivity, quality, access, or equity of the services delivered, and so on.

The most common types of small-scale research and the questions they try to address are listed in Table 2.

**Table 2:** Examples of small-scale research questions

**Common small-scale research examples**	**Research questions**
Service quality monitoring	Are we delivering a service that meets the required quality standards? What are the reasons for poor surgical outcomes (for example, cataract) in our setting? How could a particular medical or surgical service outcome be improved?
Service coverage and equity analysis	Which group(s) of people are accessing our services (by gender, socio-economic status, location, or disability)? Why are some groups not accessing our services or are not being reached by service delivery? What can be done to improve equitable access to our services?
Health worker feedback surveys	How satisfied are health workers in their work environment or management system? What is the opinion of health workers about a particular intervention?
Client satisfaction surveys	Are we meeting the needs of the community we are serving? How satisfied are our clients with the service being delivered?
Feasibility studies	What are the logistical feasibility, degree of acceptability, and costs of implementing a new health care intervention or the scaling-up of an existing intervention?
Pilot studies	Does a particular tool, process, or intervention work in the way that it is intended?

Our IMPiRE pilot study was conceived because presbyopia management services that can address the need of rural communities in Ethiopia are lacking. We wanted to answer the question: “How feasible and acceptable is the integrated management of presbyopia by primary health care workers in rural Ethiopia?”

## Data collection and synthesis

By design, small-scale research data collection can be done with limited human resources, time, and money. Data that are gathered from facility-based services and resources as part of the routine health information system can be synthesised to answer various small-scale research questions related to service access, quality, and equity. Facility-based data, if analysed and interpreted appropriately, has the potential to provide ongoing evidence on service coverage and equitable utilisation, and to do so more efficiently than expensive population-based surveys.^3^ For instance, disaggregating data by gender, location, socioeconomic indicators, and disability status can help to quickly paint a picture of who is accessing the services and whether the eye care needs of the community are being met equitably. This indicates whether progress is being made towards achieving universal eye health coverage. Service quality monitoring data, such as for cataract surgery or diabetic eye care, can easily be incorporated into routine facility-based data collection without the need for additional resources.

One of the strengths of small-scale research is the flexibility of its design. Data can be collected in a way that is appropriate to the context, but still be systematic so that it can support reliable interpretation. Like any research, different quantitative and qualitative methods can be used in small-scale research.

Primary data can be systematically collected from small groups of people through observation or interviews embedded within clinical and community-based activities. For instance, interviews or focus group discussions can be used in patient satisfaction surveys or to collect feedback on the experience of health workers in a specific programme. In the IMPiRE project, we directly observed presbyopia service delivery by a PHC worker and conducted in-depth interviews with community members and health service mangers to collect data on feasibility and acceptability.

“Primary data can be systematically collected from small groups of people through observation or interviews embedded within clinical and community-based activities.”

Secondary data can be systematically collected from randomly selected medical records to assess the prognosis of a specific intervention in a particular clinical setting.

**Figure:**
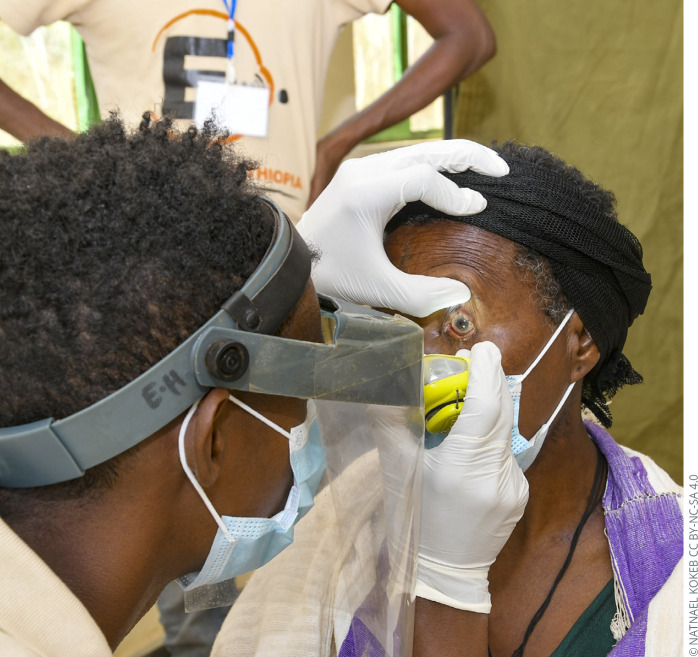
Collecting data through clinical investigations. ethiopia

## Ethics

As with any other type of research, ethics should be at the centre of small-scale research. Adherence to the ethical standards and requirements of the setting is paramount. Anonymity, confidentiality, and voluntary participation should be strictly maintained while reviewing medical records and engaging with patients and other vulnerable groups.

## Data interpretation

A healthy dose of scepticism is required when interpreting all research data, but more so when carrying out small-scale research. Findings should be interpreted with caution, as the study purpose is more concerned with improving a service or addressing contextual challenges through manageable recommendations, than with measuring the impact through statistical or monetary indicators. Data from small-scale research can often be analysed using easily accessible spreadsheet software and presented descriptively; provided the study is planned carefully, complex data analysis tools are not needed.

Regardless of the quality of the data presented, causality can rarely be inferred from small-scale studies, as sample sizes are usually too small to produce statistically significant quantitative results. Controlling for different variables and confounders are likewise challenging.

The peer review process is an integral part of research data interpretation. However, small-scale research will often not have a chance for rigorous debate and review from a wider readership through a publication process. On the other hand, small-scale research can benefit from ‘collaborative review’ – where partners such as health care managers, health workers, and stakeholders, including patients and community leaders, are involved in data interpretation.

For example, our IMPiRE pilot study provided useful data that were presented to and discussed with eye health stakeholders. Its results fed into context-specific recommendations, appropriate to the pilot study districts, and led to a large-scale research proposal that would involve investigating the equitability, quality, sustainability, and impact of the integrated management of presbyopia in a low-resource setting.

Overall, small-scale research is not conducted to test theories, but primarily to benefit the organisation conducting the research or the community it is serving. Therefore, the whole process should be a learning experience for all partners involved.^1^
